# Outcomes Among Racial and Ethnic Minority Patients With Advanced Cancers in Phase 1 Trials: A Meta-Analysis

**DOI:** 10.1001/jamanetworkopen.2024.21485

**Published:** 2024-07-11

**Authors:** Sanjay Goel, Abdissa Negassa, Mohammad H. Ghalib, Imran Chaudhary, Kavita Desai, Umang Shah, Umang Swami, Bruce Cohen, Radhashree Maitra, Sridhar Mani

**Affiliations:** 1 Department of Medical Oncology, Robert Wood Johnson Medical School, New Brunswick, New Jersey; 2 Rutgers Cancer Institute of New Jersey, New Brunswick; 3 Formerly at Montefiore Medical Center, Bronx, New York; 4 Formerly at Albert Einstein College of Medicine, Bronx, New York; 5 Department of Epidemiology and Population Health, Albert Einstein College of Medicine, Bronx, New York; 6 Department of Medical Oncology, Rutgers Cancer Institute of New Jersey, New Brunswick; 7 Department of Medical Oncology, Montefiore Medical Center, Bronx, New York; 8 Department of Radiology, Albert Einstein College of Medicine, Bronx, New York; 9 Montefiore Medical Center, Bronx, New York; 10 Department of Medical Oncology, Albert Einstein College of Medicine, Bronx, New York

## Abstract

**Importance:**

Patients from racial and ethnic minority groups (eg, Asian, Hispanic, and non-Hispanic Black patients) have low representation in clinical trials, especially in phase 1 trials in cancer. These trials represent valuable options for patients with advanced cancer who experience disease progression with standard therapy.

**Objective:**

To determine whether the benefit of enrollment to phase 1 cancer trials extends to Asian, Hispanic, and non-Hispanic Black patients as much as it does for non-Hispanic White patients.

**Data Sources:**

Patient records at a single institution from January 1999 to December 2016 were reviewed. Treatment-related responses, toxic effects, and deaths were recorded.

**Study Selection:**

All phase 1 studies were included.

**Data Extraction and Synthesis:**

Data underwent independent extraction by multiple observers following the Preferred Reporting Items for Systematic Reviews and Meta-analyses (PRISMA) reporting guideline.

**Main Outcomes and Measures:**

The primary outcome was overall survival (OS), assessed using univariate and multivariable time-to-event analyses.

**Results:**

A total of 738 patients (median [range], 60 [22-93] years; 467 [63.3] female) including 197 Hispanic patients (26.7%), 238 non-Hispanic Black patients (32.2%), and 282 non-Hispanic White patients (38.2%), were enrolled in 64 phase 1 trials, including 33 cytotoxic trials (51.5%), 21 biologic trials (32.8%), and 10 combined therapy trials (15.6%). The primary cancer diagnoses were colorectal (187 patients [25.3%]), ovarian (141 patients [19.1%]), lung (58 patients [7.9%]), uterine (49 patients [6.6%]), and breast (41 patients [5.6%]). Patients underwent a median (range) of 3 (0-13) therapies prior to trial enrollment. Among 558 patients evaluated for response, the clinical benefit rate (ie, stable disease plus response rates) was 49.1%, and the overall response rate was 6.5%. Grade 3 or 4 nonhematological toxic effects were observed in 27.8% (95% CI, 24.6%-31.3%) of patients and grade 3 or 4 hematological toxic effects were observed in 19.7% (95% CI, 17.0%-22.8%) of patients. The treatment-related mortality rate was 0.9% (95% CI, 0.4%-1.9%). Median OS was 9.6 (95% CI, 8.2-11.0) months among Hispanic patients, 8.3 (95% CI, 6.7-10.4) months among non-Hispanic Black patients, and 9.8 (95% CI, 8.5-11.4) months among non-Hispanic White patients (*P* = .13). In a multivariable analysis, age older than 60 years, Eastern Cooperative Oncology Group performance status score of 2 or greater, more than 2 metastatic sites, lactate dehydrogenase grade 1 or 2, grade 2 or greater low albumin, grade 1 or greater total bilirubin, and grade 2 or greater anemia were associated with worse prognosis, whereas leukocytosis greater than grade 1 was associated with better OS.

**Conclusions and Relevance:**

In this meta-analysis assessing outcomes in phase 1 cancer trials among patients from racial and ethnic minority groups, Hispanic and non-Hispanic Black patients had benefits similar to those of non-Hispanic White patients.

## Key Points

**Question:**

Do outcomes of Hispanic and non-Hispanic Black patients differ from those of non-Hispanic White patients in prospective phase 1 cancer clinical trials?

**Findings:**

This meta-analysis of 738 patients in 64 phase 1 trials at a single institution found a similar overall survival among patients of all races and ethnicity. In a multivariable model, several clinical variables were associated with outcomes, while race and ethnicity were not.

**Meaning:**

These findings suggest that despite low enrollment of Hispanic and non-Hispanic Black patients to Phase 1 cancer trials, their outcomes were comparable with non-Hispanic White patients.

## Teaser

This meta-analysis of phase 1 trial data from a single institution assesses whether Asian, Hispanic, and non-Hispanic Black trial participants with advanced cancer experience similar response outcomes as non-Hispanic White trial participants.

## Introduction

Phase 1 clinical trials are critical for developing novel antineoplastic drugs. They are typically offered to patients with advanced cancer who experience cancer progression with standard therapies. The primary objective of a phase 1 trial is to evaluate the dose-limiting toxicity, maximum tolerated dose, pharmacokinetics, and pharmacodynamic profiles of the drug. However, secondary objectives, such as overall response rate (ORR; calculated as partial response [PR] plus complete response [CR]) and clinical benefit rate (CBR; calculated as stable disease plus ORR), are equally important end points. In fact, multiple surveys point to the fact that almost every clinician and patient volunteered for a phase 1 trial with the objective of a clinical response,^1,2^ in addition to altruistic motive.^3^ A review of published phase 1 trials reported ORRs ranging from 4% to 12.2%,^4,5,6,7,8,9,10,11^ and CBRs ranging from 24% to 53%.^6,7,8,9,10,11^

The selection of patients for phase 1 trials remains challenging. Widely accepted eligibility criteria included a life expectancy greater than 3 months, Eastern Cooperative Oncology Group performance status (PS) score of 0 to 1 (Karnofsky PS, ≥80%), and organ function within reference ranges. Despite a careful protocol defining the selection of patients, a considerable number of patients die before the 12-week mark. However, such criteria do not effectively estimate overall survival (OS). To improve patient selection, several studies have established prognostic factors and scoring systems to better estimate OS. In all studies, multivariable analysis consistently identified lactate dehydrogenase (LDH), PS, albumin level, number of metastatic sites, platelet count, hemoglobin, lymphocyte count, and serum sodium as important prognostic variables.^5,6,7,8,11,12,13,14,15,16,17,18,19,20,21^

There is a disconcerting disparity between the racial and ethnic distribution of patients undergoing cancer therapy and those enrolled in clinical trials. There is well-established literature that non-Hispanic White patients account for more than 90% of all cancer clinical trial accruals,^22,23,24,25^ while constituting only 59.3% of the US population.^26^ More recently, as the demographics of the US have shifted, there is a trend toward a higher enrollment of patients from racial and ethnic minority groups, such as Asian, Hispanic, and non-Hispanic Black patients; however, enrollment as a share of the patient population remains low.^27^ In a large retrospective review of more than 200 trials, Hispanic and non-Hispanic Black patients represented only 6.2% and 7.9% of enrollment, respectively.^28^ A further disparate rate of non-Hispanic Black participation of 2% was reported by a different group of investigators.^29^

Regardless of recent trends, there are limited data on the enrollment of patients from racial and ethnic minority groups in phase 1 cancer trials, and to our knowledge, no study has evaluated the associations of race and ethnicity with OS in phase 1 trials. The Montefiore Einstein Cancer Center (MECC) is a National Cancer Institute (NCI)–Designated Comprehensive Cancer Center in Bronx County, New York. Bronx County is one of the country’s poorest counties and is home to one of the most ethnically and racially diverse populations. Per the 2020 census, Hispanic individuals constituted 54.8% of the population, non-Hispanic Black individuals represented 28.5%, and non-Hispanic White individuals accounted for 8.7%.^30^ Cancer centers represent one of the largest enrollers of patients from racial and ethnic minority groups in cancer clinical trials. In this study, we performed a retrospective meta-analysis of 738 patients enrolled in phase 1 trials at MECC between January 1999 and December 2016. This study aimed to characterize the clinical outcomes of our large and racially and ethnically diverse patient population treated in phase 1 trials, as well as among different races and ethnicities, that is, to explore disparities in clinical outcomes.

## Methods

### Patient Selection

The selection of patients and clinical trials followed the Preferred Reporting Items for Systematic Reviews and Meta-analyses (PRISMA) reporting guideline for this meta-analysis. Without exception, all phase 1 clinical trials conducted at MECC between January 1999 and December 2016 were included. All patients who signed informed consent and received at least 1 dose of clinical trial study medication were included. Information on each individual patient was manually collected and included demographics (age, sex, race and ethnicity), PS, number of metastases, number of prior therapies, hematologic and chemistry laboratory parameters, start and end dates of the study, response, toxic effect grades, date last seen, and date of death. Race and ethnicity were self-reported as Hispanic or non-Hispanic ethnicity and Asian, Black, White, or other (eg, American Indian or Alaska Native or Pacific Islander) race. Patients who answered Hispanic as race or ethnicity were included in the Hispanic group for this analysis. Given that all enrolled patients were included, there was no risk of bias in this aspect. Responses were determined by the World Health Organization^31^ and later by the Response Evaluation Criteria in Solid Tumors (RECIST) version at the time of the study.^32,33^ Toxic effects were assessed using the NCI Common Terminology Criteria for Adverse Events criteria, version corresponding to the year of the phase 1 study.^34^ OS was computed from the time of the first dose of the study drug and the date of death from any cause or last contact. Every study was open to patient enrollment after obtaining appropriate ethics approval from the Montefiore Einstein institutional review board. A separate protocol was not prepared for this study.

### Statistical Analysis

Descriptive summaries, such as median (range) for continuous variables and frequency (percentage) for categorical or discrete variables, were used to present baseline patient characteristics and key safety and efficacy outcomes. Response rates and associated 95% CIs (exact in the case of small numbers) have been provided. Missing data were handled using multiple imputations. Specifically, using the algorithm of full conditional specification enabled the incorporation of the extra variability induced by imputation.^35^ Essentially, rather than imputing a single value to study participants with missing covariates, we generated 50. For each realization, the corresponding set of complete data was analyzed in a standard manner, and the results were pooled using a set of rules proposed by Rubin.^36^ The imputation method regressed each covariate with a missing value using an a priori specified set of covariates and the outcome of interest. Random draws were obtained from the posterior predictive conditional distribution. An assumption in multiple imputations is that it is missing at random,^36^ which implies that the probability of missingness could depend on observed data but not on missing data values. In our database, missing at random was a reasonable assumption.

Survival experience with respect to baseline characteristics is presented as Kaplan-Meier survival curves, and, whenever possible, median survival time along with its 95% CI is provided. The association between individual baseline characteristics and survival was assessed using log-rank statistics. In modeling survival, the model assumption of proportional hazards was assessed with plots of Schoenfeld residuals vs time and accompanying formal test.^37^ We used a weighted Cox regression model^38^ to accommodate the possibility of violation of the proportional hazards assumption with respect to some of the baseline characteristics. The results were presented as point estimates or mean hazard ratios (HRs) and associated 95% CIs.

Patients were considered evaluable for response if they received the minimum number of cycles as prescribed by the protocol to perform a formal radiographic response evaluation. Response was classified per the criteria specified in the protocol as progression of disease, stable disease, PR, or CR. Patients who had a decrease in the tumor measurement of 25% to 50% per RECIST 1.1 were classified as having minor response. Patients for whom the study drug was discontinued for clinical progression even before a formal radiographic analysis were classified as having experienced progression of disease.

Statistical significance was claimed at 2-sided *P* < .05. All analyses were performed using the SAS software version 9.4 (SAS Institute) or R software version 4.0 (R Project for Statistical Computing). Data were analyzed between August 2023 and April 2024.

## Results

### Patient Clinical Characteristics and Clinical Trial Details

The meta-analysis included 64 phase 1 trials with 738 patients (median [range], 60 [22-93] years; 467 [63.3] female) (Table 1). Most patients (482 patients [65.3%]) were younger than 65 years and had a good PS (1 or better: 688 patients [93.2%]). By race and ethnicity, 20 patients (2.7%) were Asian, 197 patients (26.7%) were Hispanic, 238 patients (32.2%) were non-Hispanic Black, 282 patients (38.2%) were non-Hispanic White, and 1 patient (0.1%) identified as other race or ethnicity, reflecting the diversity of the patient population. The enrollment breakdown by race and ethnicity by time showed no statistically significant differences (eTable 1 in Supplement 1). As is common in major phase 1 cancer clinical trials, gastrointestinal cancers constituted the most frequent diagnosis, with colorectal cancer contributing 187 patients (25.3%), with contributions from pancreatic, esophagus or gastric, hepatobiliary, and hepatocellular cancers (Table 2). Another unique attribute of our population is the high number of women with gynecologic cancers, including ovarian or fallopian tube (141 patients [19.1%]) and endometrial and cervical cancers. This reflects the close collaboration between the various departments at the cancer center. Patients with lung (58 patients [7.9%]), breast (41 patients [5.6%]), and prostate (39 patients [5.2%]) cancer each represented 5% to 8% of the total study population. By therapy, trials included 33 assessing cytotoxic drugs (51.5%), 21 assessing biologic drugs (32.8%), and 10 assessing combined therapy (15.6%).

**Table 1.** Patient Characteristics at Baseline

Characteristic	Patients, No. (%) (N = 738)
Sex	
Male	271 (36.7)
Female	467 (63.3)
Unknown	0
Age, y	
Median (range)	60 (22-93)
<65 y	482 (65.3)
≥65 y	245 (33.2)
Unknown	11 (1.5)
Performance status	
0-1	688 (93.2)
2	33 (4.5)
Unknown	17 (2.3)
Race	
Asian	20 (2.7)
Hispanic	197 (26.7)
Non-Hispanic Black	238 (32.2)
Non-Hispanic White	282 (38.2)
Other^a^	1 (0.1)
Prior therapies	
Median (range)	3 (0-13)
0-2	318 (43.1)
≥3	399 (54.1)
Unknown	21 (2.8)
Metastatic sites, No.	
Median (range)	2 (0-7)
0	9 (1.2)
1-2	393 (53.3)
≥3	309 (41.9)
Unknown	27 (3.7)
Metastatic specific sites	
Liver	315 (42.7)
Lung	321 (43.5)
Bone	141 (19.1)
Unknown	27 (3.7)
Albumin, g/dL	
Median (range)	3.9 (1.8-5.4)
<3.5	151 (20.5)
≥3.5	577 (78.2)
Unknown	10 (1.4)
LDH, U/L	
Median (range)	240 (101-4937)
<240	350 (47.4)
≥240	351 (47.6)
Unknown	37 (5)
Hemoglobin, g/dL	
Median (range)	10.8 (6-16.2)
<10.5	324 (43.9)
≥10.5	404 (54.7)
Unknown	10 (1.4)
WBC, cells/μL	
Median (range)	5.2 (0.4-73.2)
≤10 500	660 (89.4)
>10 5000	66 (8.9)
Unknown	12 (1.6)
Platelets, ×10^3^/µL	
Median (range)	227 (5-1231)
≤400	657 (89)
>400	71 (9.6)
Unknown	10 (1.4)

Abbreviations: LDH, lactate dehydrogenase; WBC, white blood cell.

SI conversion factors: To convert albumin and hemoglobin to grams per liter, multiply by 10; LDH to microkatals per liter, multiply by 0.0167; platelets to ×10^9^/L, multiply by 1; WBC to ×10^9^/L, multiply by 0.001.

^a^ Includes American Indian or Alaska Native and Pacific Islander.

**Table 2.** Primary Tumor Sites

Primary tumor site	Participants, No. (%) (N = 738)
Colorectal	187 (25.3)
Ovarian or fallopian tube	141 (19.1)
Lung	58 (7.9)
Endometrial or uterine	49 (6.6)
Breast	41 (5.6)
Prostate	39 (5.3)
Esophageal or gastric	37 (5.0)
Cervical	30 (4.1)
Head and neck	22 (3.0)
Pancreatic	21 (2.8)
Renal	20 (2.7)
Bladder	15 (2.0)
ACUP	13 (1.8)
Hepatobiliary	9 (1.2)
Sarcoma	8 (1.1)
Anal	7 (0.9)
Liver	6 (0.8)
Melanoma	6 (0.8)
Lymphoma	3 (0.4)
Other	26 (3.5)

Abbreviation: ACUP, adenocarcinoma of unknown primary.

### Patient Characteristics

Among 738 patients enrolled, the median (range) number of prior therapies was 3 (0-13). For example, a trial incorporated gemcitabine and was considered a reasonable first-line therapy for patients with metastatic pancreatic ductal adenocarcinoma when it was the only drug approved for these patients.^39^ The median (range) number of metastatic sites was 2 (0-7), with the most common sites being the liver (315 patients [42.7%]) and lung (321 patients [43.5%]). Surprisingly, 141 patients (19.1%) had bone metastases at the time of enrollment. Laboratory analyses of important baseline characteristics, including albumin, LDH, hemoglobin, total white blood cell (WBC) count, and platelets, are listed in Table 1.

### Efficacy Outcomes

Among 738 patients enrolled, 585 (79.3%) were evaluable for response assessment, similar to the published reports of phase 1 trials. Among them, 6 patients (1%; 95% CI, 0.42%-2.3%) experienced a CR, and 32 patients (5.5%; 95% CI, 3.8%-7.7%) had a PR (eTable 2 in Supplement 1). While a minor response was not a criterion-recognized entity, 6 patients (1%; 95% CI, 0.42%-2.3%) had a reduction of 25.0% to 49.9%, and were classified as such. Stable disease was observed in 41.5% (95% CI, 37.5%-45.7%) of patients, with a total clinical benefit rate of 49.1% (95% CI, 44.9%-53.2%). The overall response rate was 6.5% (95% CI, 4.6%-8.8%). In summary, almost half of the enrolled patients who were eligible for the response assessment experienced clinical benefits.

Breaking down OS by year of enrollment, we observed a longer OS among patients enrolled in more recent years, with an OS of 12.03 (95% CI, 8.8-15.4) months for patients enrolled between 2011 and 2016, as opposed to 9.53 (95% CI, 8.4-10.6) months for those enrolled between 1999 and 2004 and 8.2 (95% CI, 6.7-9.6) months for patients enrolled between 2005 and 2010 (*P* < .001) (eTable 3 in Supplement 1). Assessing OS by type of therapy received did not yield any differences. The median OS was 9.57 (95% CI, 8.50-10.8) months for patients who received cytotoxic therapy, 8.73 (95% CI, 6.97-11.63) months for patients who received biologics, and 9.17 (95% CI, 7.5-12.2) months for patients who received combined therapy (*P* = .71) (eTable 4 in Supplement 1).

Age older than 60 years (HR, 1.25; 95% CI, 1.03-1.51); *P* = .03), PS of 2 or greater (HR 1.77; 95% CI, , *P* = .02), more than 2 metastatic sites (HR, 1.23; 95% CI, 1.01-1.50; *P* = .04), LDH grade 1 (HR, 1.28; 95% CI, 1.03-1.58; *P* = .03), LDH grade 2 (HR, 1.74; 95% CI, 1.28-2.37; *P*  < .001), hypoalbuminemia greater than grade 1 (HR, 2.46; 95% CI, 1.95-3.11; *P* < .001), total bilirubin grade 1 or greater (HR, 1.82; 95% CI, 1.10-2.99; *P* = .02), AST grade 1 or greater (HR, 1.54; 95% CI, 1.12-2.13; *P* = .008), and anemia grade 2 or greater (HR, 1.40; 95% CI, 1.04-1.88; *P* = .03) were negatively associated with OS (Table 3 and Table 4; ; eFigure in Supplement 1). Leukocytosis greater than grade 1 (HR, 0.72; 95% CI, 0.58-0.90; *P* = .003) was positively associated with OS (Table 3 and Table 4; eFigure in Supplement 1).

**Table 3.** Univariate Analysis of Factors Associated With Overall Survival

Variable	Hazard ratio (95% CI)	*P* value
Age, y		
≤60	1 [Reference]	NA
>60	1.20 (1.01-1.43)	.04
Sex		
Male	1 [Reference]	NA
Female	1.07 (0.89-1.29)	.48
Race and ethnicity		
Asian	0.79 (0.42-1.50)	.47
Hispanic	0.99 (0.80-1.23)	.94
Non-Hispanic Black	1.14 (0.92-1.42)	.22
Non-Hispanic White	1 [Reference]	NA
Performance status		
<2	1 [Reference]	NA
≥2	3.11 (2.08, 4.66)	<.001
Metastatic sites, No.		
≤2	1 [Reference]	NA
>2	1.29 (1.08-1.55)	.006
Lines of prior therapies, No.		
≤2	1 [Reference]	NA
>2	1.00 (0.84-1.20)	.98
LDH Grade		
0	1 [Reference]	NA
1	1.38 (1.14-1.69)	.001
2	2.91 (2.21-3.82)	<.002
Albumin grade		
0	1 [Reference]	NA
≥1	3.16 (2.51-3.98)	<.001
Total bilirubin grade		
0	1 [Reference]	NA
≥1	2.11 (1.25-3.58)	.005
AST grade		
0	1 [Reference]	NA
≥1	1.78 (1.42-2.24)	<.001
ALT grade		
0	1 [Reference]	NA
≥1	1.27 (0.97-1.67)	.08
WBC grade		
0	1 [Reference]	NA
≥1	0.66 (0.55-0.80)	<.001
Hemoglobin grade		
0	1[Reference]	NA
1	1.11 (0.88-1.39)	.04
2	1.82 (1.41-2.34)	<.001
Platelet grade		
0	1 [Reference]	NA
≥1	0.90 (0.70-1.16)	.43

Abbreviations: ALT, alanine aminotransferase; AST, aspartate aminotransferase; LDH, lactate dehydrogenase; NA, not applicable; WBC, white blood cell.

**Table 4.** Multivariable Analysis of Prognostic Factors for Overall Survival

Variable	Hazard ratio (95% CI)	*P* value
Age, y		
≤60	1 [Reference]	NA
>60	1.25 (1.03-1.51)	.03
Sex		
Male	1 [Reference]	NA
Female	0.98 (0.79-1.21)	.86
Race and ethnicity		
Asian	0.75 (0.34-1.65)	.47
Hispanic	0.96 (0.76-1.21)	.71
Non-Hispanic Black	1.01 (0.80-1.29)	.92
Non-Hispanic White	1 [Reference]	NA
Performance status		
<2	1 [Reference]	NA
≥2	1.77 (1.11, 2.80)	.02
Metastatic sites , No.		
≤2	1 [Reference]	NA
>2	1.23 (1.01-1.50)	.04
LDH grade		
0	1 [Reference]	NA
1	1.28 (1.03-1.58)	.03
2	1.74 (1.28-2.37)	<.001
Albumin grade		
0	1 [Reference]	NA
≥1	2.46 (1.95-3.11)	<.001
Total bilirubin grade		
0	1 [Reference]	NA
≥1	1.82 (1.10-2.99)	.02
AST grade		
0	1 [Reference]	NA
≥1	1.54 (1.12-2.13)	.008
ALT grade		
0	1 [Reference]	NA
≥1	0.76 (0.50-1.13)	.18
WBC grade		
0	1 [Reference]	NA
≥1	0.72 (0.58-0.90)	.003
Hemoglobin grade		
0	1 [Reference]	NA
1	1.07 (0.84-1.37)	.56
2	1.40 (1.04-1.88)	.03

Abbreviations: ALT, alanine aminotransferase; AST, aspartate aminotransferase; LDH, lactate dehydrogenase; NA, not applicable; WBC, white blood cell.

### Efficacy Outcomes by Race and Ethnicity

Given the low representation of patients from racial and ethnic minority groups in cancer clinical trials, particularly in phase 1, we explored the possibility of disparities in clinical outcomes by race and ethnicity. It was encouraging to observe that Hispanic and non-Hispanic Black patients experienced similar OS outcomes as their non-Hispanic White counterparts (Figure, Table 3, and Table 4; eTable 5 in Supplement 1). The median OS by race was 10.2 (95% CI, 5.0, 48.9) months for Asian patients, 9.6 (95% CI, 8.2-11.0) months for Hispanic patients, 8.3 (95% CI, 6.7-10.4) months for Black patients, and 9.8 (95% CI, 8.5-11.4) months for non-Hispanic White patients. This study was not designed to formally test disparities in clinical outcomes, and so these results need to be interpreted cautiously.

**Figure.**
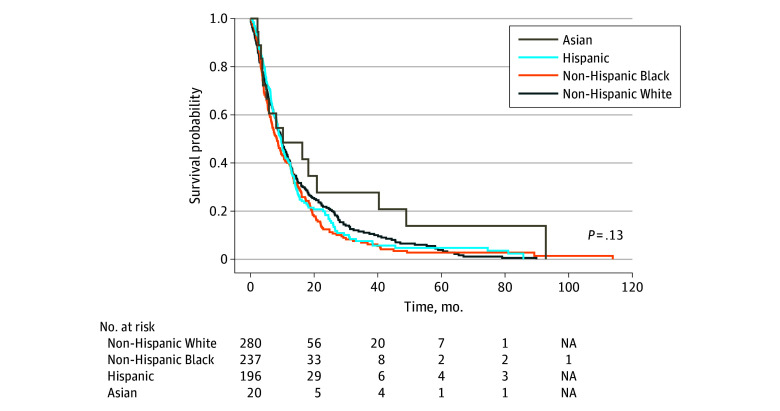
Overall Survival (OS) of All Patients by Race and Ethnicity There was no statistically significant difference in this outcome.

In terms of response and clinical benefit, there were no statistically significant differences among the racial and ethnic groups. However, the ORRs were higher among Asian patients (11.7%; 95% CI, 1.5%-36.4%) and non-Hispanic White patients (9.5%; 95% CI, 6.0%-14.0%) compared with Hispanic patients (4.1%; 95% CI, 1.5%-8.7%) and non-Hispanic Black patients (4.2%; 95% CI, 1.8%-8.1%) (*P* = .05). No such trend was noted in the CBR (eTable 6 in Supplement 1).

### Toxic Effects

All 738 patients had received at least 1 dose of study drug and were considered evaluable for toxic effects assessment. Focusing on the major toxic effects, grade 3 to 4 non-hematological toxic effects were observed in 27.8% (95% CI, 24.6%-31.3%) of patients and hematological toxic effects were observed in 19.7% (95% CI, 17.0%-22.8%) of patients. The overall treatment-related mortality rate was 0.9% (95% CI, 0.4%-1.9%). No differences were noted in the major toxic effects rate or the toxic death rate among the racial and ethnic groups.

## Discussion

This meta-analysis reports on the experiences of racial and ethnic minority patients in phase 1 cancer clinical trials and found that the clinical benefit was similar for all patients, regardless of their racial and ethnic backgrounds. The availability of new drugs with improved toxicity profiles and increased efficacy is critical in our efforts to improve the lives of patients with cancer. Drug development is a multistep process that takes years to mature and generate sufficient evidence of clinical benefits to justify regulatory approval and clinical adoption of new agents. Phase 1 dose-finding studies are the initial and crucial step in this process.

Although clinical trials form the backbone of drug development and approval, they are plagued by several limiting factors. For example, enrollment and completion of clinical trials in general and phase 1 trials in particular have underrepresentation of patients from racial and ethnic minority groups in cancer clinical trials.^22,23,24,25^ In an unrelated single-institution study, Perni et al^29^ reported a lower enrollment of non-Hispanic Black patients in phase 1 trials (2% of participants) than in phase 2 and 3 trials (4% of participants).^29^ The corresponding figures for the Hispanic participants are 2% in phase 1 trials and 5% in phase 2 and 3 trials. In the NCI experience of phase 1 trials, 8.7% of patients were non-Hispanic Black and 84.3% of patients were non-Hispanic White.^4^

It is believed that the unfair treatment of patients from racial and ethnic minority groups by the US health care system, including the infamous Tuskegee trials^40,41^ and genetic studies among American Indian populations,^42^ has led to a lower interest in clinical trial participation. However, when studied in an objective manner, the results are clear: patients from racial and ethnic minority groups remain open to and interested in learning about the opportunity to partake in trials; it simply needs to be discussed with them as an option during the consultation.^43,44^

A novel observation of this study was the similar survival rates experienced by patients of all racial and ethnic groups. The median OS was 9.6 months among Hispanic patients, 8.3 months in non-Hispanic Black patients, and 9.8 months in non-Hispanic White patients. While several recent reports have focused on phase 1 experiences among patients with cancer, most have focused on response rates. Our single-center robust database with close patient follow-up facilitated the collection of comprehensive data, including survival times. As a result, we were able to offer a window into the OS experiences of patients from different racial and ethnic backgrounds.

Similar to other reported studies, several clinical and laboratory parameters significantly affected the OS of patients who chose to participate in Phase 1 clinical trials. The single most relevant factor that determined OS was hypoalbuminemia, with patients with levels below reference range facing a 146% higher risk of death than those with levels within the reference range. Albumin is a surrogate marker for the overall health and nutritional status of individual.^45,46^ As expected, a PS at study entry of 2 or worse was also associated with worse OS. Other clinically important factors included LDH level, liver function, and anemia.

One new finding in this report was that patients with a leukocytosis experienced a more favorable outcome than those with a WBC count within reference range. This may represent a better clinical profile for overcoming myelosuppression induced by cytotoxic agents, as three-quarters of our trials included these agents. There is limited literature regarding the role of leukocytosis as a prognostic marker in patients with solid tumors. In a study of patients with colorectal cancer, preoperative leukocytosis was associated with worse postoperative outcomes.^47^ The role of total WBC count as a prognostic marker in patients with advanced renal cancer is well established as part of the International Metastatic RCC Database Consortium risk stratification.^48^ In this model, patients with a leukocytosis had a worse OS than those with a WBC count within reference range. An important distinction is that kidney cancer is treated using immune therapy–based approaches rather than myelosuppressive cytotoxic chemotherapy, unlike the experience highlighted in this study.

Other interesting observations in our study included an improvement in OS among patients who were treated more recently. Patients who were enrolled after 2010 experienced an OS of more than 1 year, unlike those who were enrolled before 2010. While one may be keen to attribute these improvements to better drugs, we must not fail to recognize the innovations and improvements in supportive care that have contributed to better patient experience and outcomes. Furthermore, greater availability of standard approved agents following discontinuation of the phase 1 trial may also contribute to enhanced OS. A 2022 report from the NCI Cancer Therapy Evaluation Program that summarized their experience with more than 13 000 patients also noted improved response rates over time.^4^

### Limitations

This study has some limitations that merit further discussion. First, despite the relatively large number of patients in the database, this was a single-institution study with a narrow scope. Second, our study spans a period of 16 years and carries the burden of heterogeneity of patients, shifting demographics, nature of drugs tested, improvement in supportive care, and rapid alterations in standard of care, with significant improvement in the OS of patients. Despite this, the results do not eliminate the fact that there is a constant need for new drugs to be tested, and a study that spans a long period offers a window into this fact. Third, immunotherapy drugs are represented only in a limited manner in our database. It is likely that phase 1 trials incorporating this class of agents will have a better outcome for enrolled patients, and as such, these data may underestimate the benefits that patients can expect when they make a decision to enroll in a phase 1 cancer clinical trial.

## Conclusions

In this meta-analysis, we present the largest multiracial and multiethnic study of phase 1 trials for patients with cancer to our knowledge. We found that enrollment of patients from racial and ethnic minority groups in such trials is feasible, and encouraged, and that the clinical benefits and outcomes are similar for all patients.

## Notes

Supplement 1. **eTable 1.** Patient Enrollment by Time and Race and Ethnicity **eTable 2.** Response Outcome Among 585 Patients Evaluable for Response **eTable 3.** Split of the Timeline of the Study into 3 Periods **eTable 4.** Therapy Received by Mechanism of Action **eTable 5.** Pairwise Comparison by Race and Ethnicity in the OS Experiences of Patients From Racial and Ethnic Minority Groups **eTable 6.** Comparison of RR and CBR by Race and Ethnicity **eFigure.** Survival Curves of Patients Based on Risk Factors

Supplement 2.
Data Sharing Statement
