# Error in Author Degree

**DOI:** 10.1001/jamanetworkopen.2024.33744

**Published:** 2024-08-20

In the Original Investigation titled “Outcomes Among Racial and Ethnic Minority Patients With Advanced Cancers in Phase 1 Trials: A Meta-Analysis,”^1^ published July 11, 2024, there was an error in the degree provided for Kavita Desai. Dr Desai has an MD. This article has been corrected.^1^
