# Supplementary material for: Outcomes Among Racial and Ethnic Minority Patients With Advanced Cancers in Phase 1 Trials: A Meta-Analysis
Source: JAMA Netw Open. 2024 Jul 11;7(7):e2421485. doi: 10.1001/jamanetworkopen.2024.21485 (PMC11240188; doi:10.1001/jamanetworkopen.2024.21485)
Supplement: Supplement 1. — eTable 1. Patient Enrollment by Time and Race and Ethnicity eTable 2. Response Outcome Among 585 Patients Evaluable for Response eTable 3. Split of the Timeline of the Study into 3 Periods eTable 4. Therapy Received by Mechanism of Action eTable 5. Pairwise Comparison by Race and Ethnicity in the OS Experiences of Patients From Racial and Ethnic Minority Groups eTable 6. Comparison of RR and CBR by Race and Ethnicity eFigure. Survival Curves of Patients Based on Risk Factors [file jamanetwopen-e2421485-s001.pdf]

Supplemental Online Content

Goel S, Negassa A, Ghalib MH, et al. Outcomes among racial and ethnic minority patients with advanced cancers in phase 1 trials: a meta-analysis. *JAMA Netw Open*. 2024;7(7):e2421485. doi:10.1001/jamanetworkopen.2024.21485

eTable 1. Patient Enrollment by Time and Race and Ethnicity
eTable 2. Response Outcome Among 585 Patients Evaluable for Response
eTable 3. Split of the Timeline of the Study into 3 Periods
eTable 4. Therapy Received by Mechanism of Action
eTable 5. Pairwise Comparison by Race and Ethnicity in the OS Experiences of Patients From Racial and Ethnic Minority Groups
eTable 6. Comparison of RR and CBR by Race and Ethnicity
eFigure. Survival Curves of Patients Based on Risk Factors

This supplemental material has been provided by the authors to give readers additional information about their work.

e Table 1

Patient enrollment[1] by time and race/ethnicity

	Year			
Race/Ethnicity	1999-2004	2005-2010	2011-2016	p-value[2]
NHW	133 (42%)	94 (39%)	53 (31%)	0.26
NHB	93 (29%)	85 (35%)	59 (34%)	
Hispanic	86 (27%)	56 (23%)	54 (31%)	
Asian	7 (2.2%)	7 (2.9%)	6 (3.5%)	

[1]n (%)
[2]Monte-Carlo resampling (~10,000)

e Table 2

Treatment response

**Treatment Response**	**n=585**	**%**
Complete Response (CR)	6	1.0
Partial Response (PR)	32	5.5
Minor Response (MR)	6	1.0
Stable Disease (SD)	243	41.5
Progressive Disease (PD)	298	50.9
Overall Response Rate (ORR) [CR+PR	38	6.5
Clinical Benefit Rate (CBR) [SD+MR +ORR]	287	49.1

e Table 3

Median OS by year of study

Year of Phase I trial	Median OS (95% CI)	P-value
1999-2004	9.53 (8.40, 10.56)	<0.001
2005-2010	8.20 (6.70 , 9.63)	
2011-2016	12.03 (8.83 - 15.43)	

e Table 4

Median OS by type of therapy

Type of therapy	Median OS (95% CI)	P-value
Cytotoxic	9.57 (8.50 ,– 10.8)	0.71
Biologic	8.73 (6.97 , 11.63)	
Combined	9.17 (7.5 , 12.20)	

© 2024 Goel S et al. *JAMA Network Open.*

e Table 5

Pairwise comparison by race/ethnicity in OS

	$X^2_{(1)}$	*p*-value	*p*-value Adjusted*
NHB vs Hispanic	0.22	0.64	0.94
NHB vs Asian	0.49	0.49	0.94
Hispanic vs Asian	0.32	0.58	0.94

**p*-value adjusted for multiple testing using the Benjamini and Hochberg approach. The contrasts of each racial group with NHW is already shown in Table 4.

e Table 6

Response Rate (RR) and Clinical Benefit Rate (CBR) by race/ethnicity

Response[1]	White (n=232)	Black (n=190)	Hispanic (n=146)	Asian (n=17)	*p*-value[2]
ORR	22 (9.5%)	8 (4.2%)	6 (4.1%)	2 (12%)	0.054
CBR	126 (54%)	90 (47%)	66 (45%)	5 (29%)	0.10

[1]n (%)
[2]Pearson’s Chi-squared test; Fisher’s exact test

© 2024 Goel S et al. *JAMA Network Open.*

SUPPLEMENTARY FIGURE

eFigure 1a

Kaplan-Meier Curves for OS by LDH

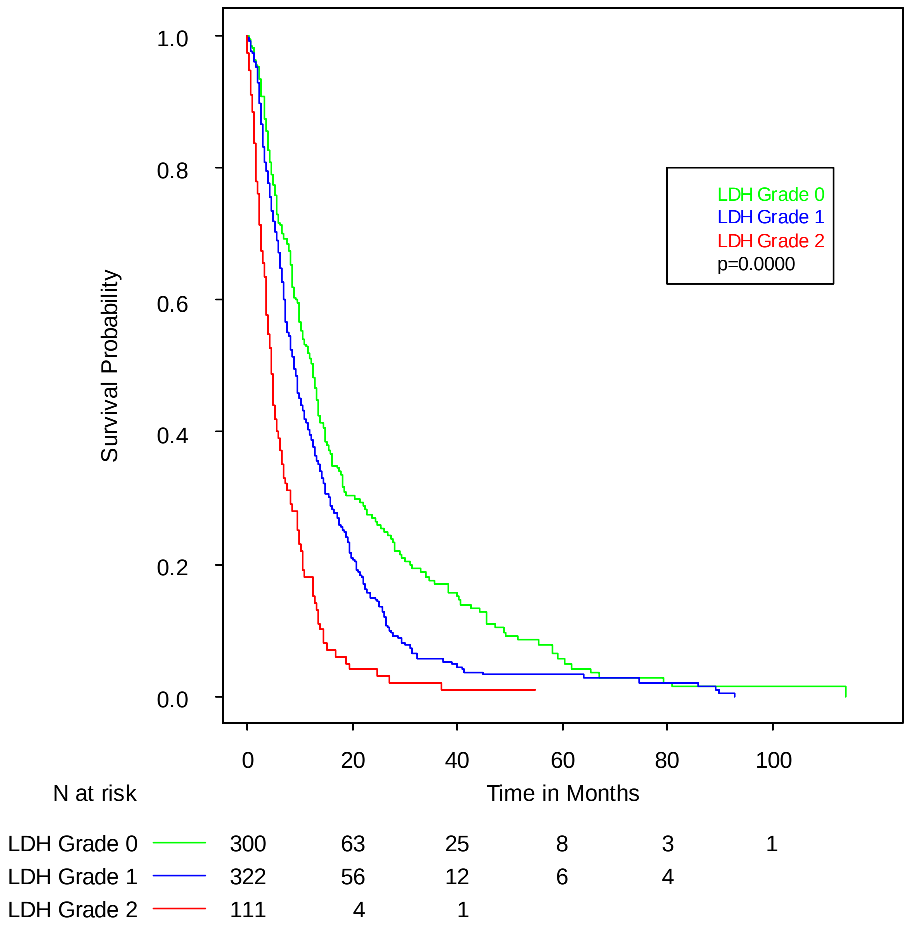

© 2024 Goel S et al. *JAMA Network Open.*

eFigure 1b

Kaplan-Meier Curves for OS by Albumin

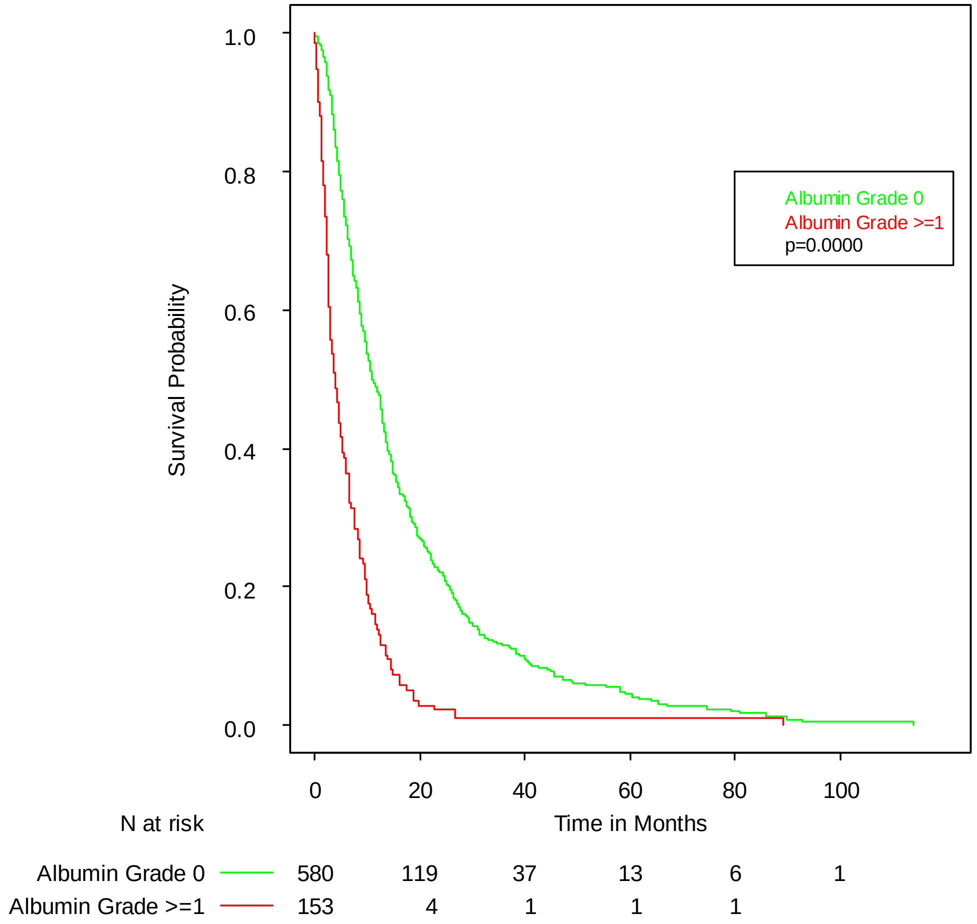

© 2024 Goel S et al. *JAMA Network Open.*

eFigure 1c

Kaplan-Meier Curves for OS by WBC

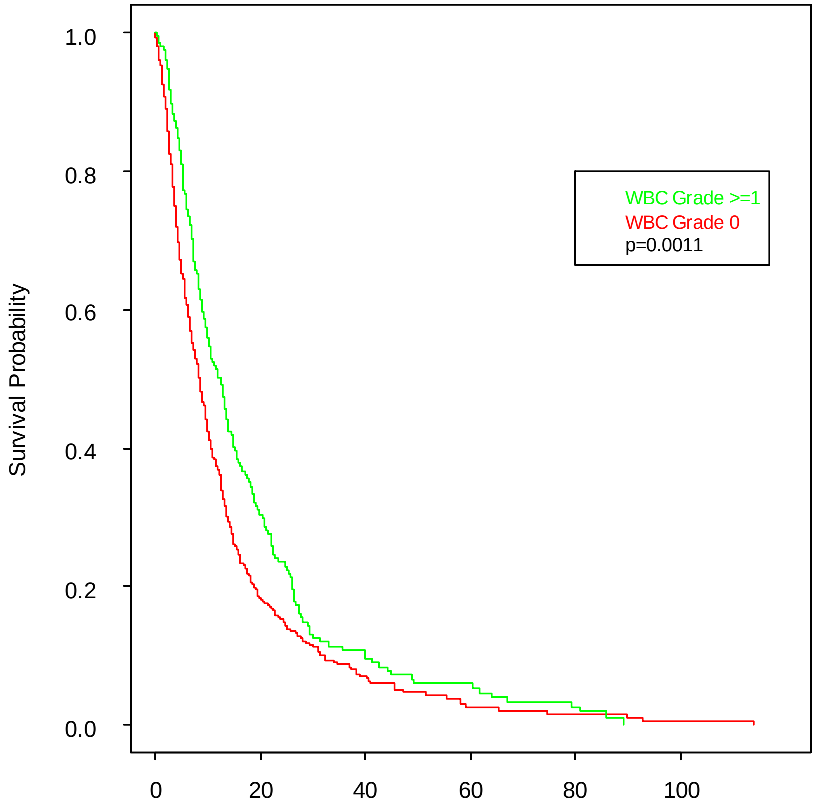

N at risk	0	20	40	60	80	100
WBC Grade >=1	216	53	17	9	4	
WBC Grade 0	517	70	21	5	3	1

© 2024 Goel S et al. *JAMA Network Open.*

PRISMA 2020 flow diagram for new systematic reviews which included searches of databases and registers only

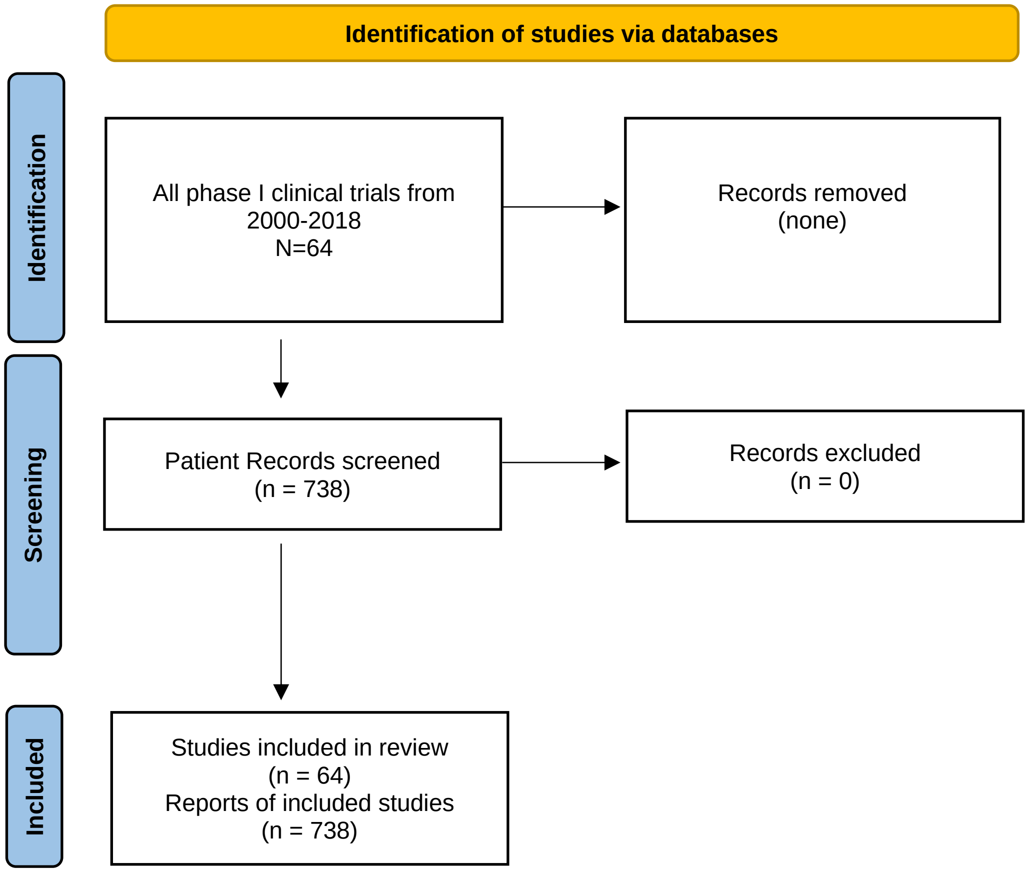

*Consider, if feasible to do so, reporting the number of records identified from each database or register searched (rather than the total number across all databases/registers).

**If automation tools were used, indicate how many records were excluded by a human and how many were excluded by automation tools.

From: Page MJ, McKenzie JE, Bossuyt PM, Boutron I, Hoffmann TC, Mulrow CD, et al. The PRISMA 2020 statement: an updated guideline for reporting systematic reviews. BMJ 2021;372:n71. doi: 10.1136/bmj.n71

For more information, visit: http://www.prisma-statement.org/

© 2024 Goel S et al. *JAMA Network Open.*
