# Supplementary material for: Outcomes Among Racial and Ethnic Minority Patients With Advanced Cancers in Phase 1 Trials: A Meta-Analysis
Source: JAMA Netw Open. 2024 Jul 11;7(7):e2421485. doi: 10.1001/jamanetworkopen.2024.21485 (PMC11240188; doi:10.1001/jamanetworkopen.2024.21485)
Supplement: Supplement 2. — Data Sharing Statement [file jamanetwopen-e2421485-s002.pdf]

Data Sharing Statement

Goel. Outcomes Among Racial and Ethnic Minority Patients With Advanced Cancers in Phase 1 Trials. *JAMA Netw Open*. Published July 11, 2024.
doi:10.1001/jamanetworkopen.2024.21485

Data
Data available: Yes
Data types: Deidentified participant data, Other (please specify)
Additional Information: Can provide basic demographic and outcome data
How to access data: sanjay.goel@rutgers.edu
When available: With publication

Supporting Documents
Document types: Other (please specify)
Additional Information: Basic data with outcomes
How to access documents: sanjay.goel@rutgers.edu
When available: With publication

Additional Information
Who can access the data: Anyone requesting data
Types of analyses: Any other analysis
Mechanisms of data availability: Signed DUA
Any additional restrictions: We are unable to share the data via a publicly available system due to privacy concerns. If an individual reader wishes to obtain information on a specific aspect of the data, we can reach out to our institution policy and attempt to obtain consent to share patient level data. We can share the statistical analysis information.
